# Experimental traumatic brain injury

**DOI:** 10.1186/2040-7378-2-16

**Published:** 2010-08-13

**Authors:** Christiane Albert-Weissenberger, Anna-Leena Sirén

**Affiliations:** 1 University of Würzburg, Department of Neurology, Würzburg, Germany; 2 University of Würzburg, Department of Neurosurgery, Würzburg, Germany

## Abstract

Traumatic brain injury, a leading cause of death and disability, is a result of an outside force causing mechanical disruption of brain tissue and delayed pathogenic events which collectively exacerbate the injury. These pathogenic injury processes are poorly understood and accordingly no effective neuroprotective treatment is available so far. Experimental models are essential for further clarification of the highly complex pathology of traumatic brain injury towards the development of novel treatments. Among the rodent models of traumatic brain injury the most commonly used are the weight-drop, the fluid percussion, and the cortical contusion injury models. As the entire spectrum of events that might occur in traumatic brain injury cannot be covered by one single rodent model, the design and choice of a specific model represents a major challenge for neuroscientists. This review summarizes and evaluates the strengths and weaknesses of the currently available rodent models for traumatic brain injury.

## 

Traumatic brain injury (TBI) is a result of an outside force causing immediate mechanical disruption of brain tissue and delayed pathogenic events which collectively mediate widespread neurodegeneration (reviewed by [1]). It is a heterogeneous disorder that can vary in the type of brain injury, distribution of brain damage and mechanisms of damage (Table 1). The primary damage of brain tissue can be diffuse or focal whereby the circumstances of injury determine the relative degree to which diffuse and focal trauma develops. Primary injury caused by direct impact to the head is considered to be largely focal, and results in cortical contusion, vascular injury and hemorrhages accompanied by ischemia. In contrast, diffuse brain injury characterized by diffuse axonal injury is caused by acceleration/deceleration forces. Depending upon the nature of primary injury, various cell responses are triggered that can exacerbate the injury. To date, these secondary injury processes are poorly understood.

**Table 1:** Leading clinical causes and types of TBI in the United States 2002 - 2006 [2]

Cause	Percentage of		Physical mechanism	Primary brain injury
	TBI	TBI-related deaths		
Falls	35.2%	18.9%	impact resulting in the acceleration of the head and brain [125,126]	closed head injury

Road traffic accidents	17.3%	31.8%	impact and acceleration of the head and brain [125,127]	closed head injury

Struck by/against events	16.5%	0.7%	impact resulting in the acceleration of the head and brain [125,126]	closed head injury

TBI remains a leading cause of death and disability in the industralized countries [2,3] and represents a growing health problem also in the developing countries [4-7]; therefore even a modest outcome improvement could have major public health implications. As the immediate cell death resulting from the initial impact on the brain tissue is irreversible, treatments focus on interruption or inhibition of the secondary injury cascades expanding this primary injury. Nonetheless, no effective neuroprotective treatment is available so far [8-11]. The use of animal models is essential for better understanding of the secondary injury processes and for the development on novel therapies. Although large animal models may be necessary to investigate specific aspects of TBI, rodents (mice and rats) have emerged as the most commonly used species (for a review see [12]), since they are easily available to many researchers, normative data for a wide range of physiological and behavioral variables in rodents are well documented and transgenic technologies allow the generation of rodent lines with specific genetic alterations. A number of mouse and rat models have been developed to induce brain trauma. Of these the most commonly used are weight-drop injury, fluid percussion injury (FPI), and cortical contusion injury (CCI). However, the entire spectrum of events that might occur in TBI cannot be covered by one single rodent model. Therefore, this review evaluates the strengths and weaknesses of the currently available rodent models for TBI (Table 2).

**Table 2:** Experimental rodent models of closed-head injury

Model	Species	Injury	Strengths	Weaknesses
**Weight-drop models**				
Feeney's weight-drop Shohami's weight-drop Marmarou's weight-drop	rat [22] rat [14], mouse [15] rat [16,17], mouse [43]	predominantly focal predominantly focal predominantly diffuse	injury mechanism and inflicted injury is close to human TBI severity of injury can be adjusted well characterized neuroscoring immediately after injury allows randomization	high mortality rate due to apnea and skull fractures not highly reproducible

**FP models**				
MFP LFP	rat [46,47] rat [48], mouse [49]	mixed mixed	severity of injury can be adjusted inflicted injury is highly reproducible within one laboratory	requires craniotomy that may compensate for ICP increases no immediate post-injury neuroscoring possible inflicted injury is variable between laboratories high mortality rate due to apnea

**CCI**	rat [72], mouse [73]	predominantly focal	severity of injury can be adjusted inflicted injury is highly reproducible	requires craniotomy no immediate post-injury neuroscoring

**Cryogenic brain lesion**	rat [92], mouse [93]	focal	severity of injury can be adjusted inflicted injury is highly reproducible and easily quantifiable	mimics only conditionally human TBI

## Weight-drop models

The weight-drop models use the gravitational forces of a free falling weight to produce a largely focal [13-15] or diffuse [16-19] brain injury. The impact of the free falling weight is delivered to the exposed skull in rat [14] and mouse [20] or the intact dura in rat [21,22]. When the impact is delivered to the exposed skull, generally soft tips, e.g. silicon-covered [15] reduce the risk of skull fractures. For inducing focal brain injury, the animals are placed on non-flexible platforms in order to minimize dissipation of energy [13-15]. In contrast, crucial for inducing a diffuse brain injury is an impact widely distributed over the skull and the use of flexible platforms allowing the head to accelerate, e.g. foam-type platforms [16,17,19] or platforms with elastic springs [18]. The severity of head trauma can be varied by using different weights and/or heights of the weight-drop. The high mortality rate due to apnea can be reduced by early respiratory support and the usage of animals with a certain age and weight [16,17].

### Feeney's weight-drop model

Typically this rat model in which an impact is delivered to the intact dura [21,22] results in a cortical contusion with hemorrhage [23] and damage of the blood-brain barrier [24,25]. Inflammatory processes lead to activation of microglia and astrocytes, activation of the complement system and invasion of neutrophils and macrophages [22,23,25-31]. Delayed microcirculatory disturbances and cortical spreading depression [32] have also been reported in this model. The pattern of posttraumatic cell death depends on the severity of impact [33]. Although the primary injury is largely focal, diffusely distributed axonal injury has been observed in the neuropil of the cortical lesion [23].

### Shohami's weight-drop model

Shapira et al. and Chen et al., later introduced a model for closed-head injury using a weight-drop impact to one side of the unprotected skull in rat [14] and mouse [15,20], respectively. The injury severity in this model is dependent on the mass and falling height of the weight used. Thus, heavier weights and/or increased falling height produces an ipsilateral cortical brain contusion and blood-brain barrier disruption followed by brain edema, activation of the complement system, cell death evolving over time from the contusion site and invasion of inflammatory cells [13,15,23,34-37]. A modified model using lighter weights and/or shorter fall heights resulted in a concussive-like brain injury, bilateral cell loss, short duration of brain edema and long-lasting cognitive deficits [23]. Moreover, bilateral diffuse brain damage, cell death (bilateral and beneath the impact site), and inflammatory responses were reported [38-41]. In general mild weight-drop injuries are associated with a diffuse injury pattern whereas more severe weight-drop injuries produce a focal contusion. A disadvantage of the weight-drop model is the high variability of the injury severity. A major advantage of this model is that it can be quickly performed under gas-anesthesia and thus allows neurological scoring immediately after injury [15,20]. Thus clinically relevant randomization of animals into the various treatment groups is possible.

### Marmarou's weight-drop model (Impact acceleration model)

To model "whole head" motion resulting in a diffuse brain injury, Marmarou et al. [16,17] allows the head to accelerate at impact. Depending on the severity of injury, the induced brain injury results in hemorrhages, neuronal cell death, astrogliosis, diffuse axonal injury, and cytotoxic brain edema [17,23,26,42,43]. This impact acceleration model using a weight-drop is a useful model for investigating diffuse brain injuries ranging from mild to severe.

Taken together, weight-drop models provide a straightforward way to assess brain injuries close to the clinical conditions ranging from focal to diffuse brain injuries.

## Fluid percussion injury models

Fluid percussion injury (FPI) models produce brain injury by rapidly injecting fluid volumes onto the intact dural surface through a craniotomy. The craniotomy is made either centrally (CFP, MFP), over the sagittal suture midway between bregma and lambda, or laterally (LFP), over the parietal cortex. Graded levels of injury severity can be achieved by adjusting the force of the fluid pressure pulse. Like in various other TBI models, a high mortality rate due to apnea is evident [44,45].

The central (CFP) and lateral (LFP) fluid percussion injury models were adapted to rats in 1987 [46,47] and in 1989 [48,49], respectively. These models produce a mixed type of brain injury. Traumatic pathology includes cortical contusion, hemorrhage and a cytotoxic and/or vasogenic brain edema either typically bilateral for CFP injury or ipsilateral for LFP injury [23,26,50]. The delayed progression of brain damage is accompanied by astrogliosis, diffuse axonal injury, inflammatory events, cortical spreading depression and neurodegeneration [23,26,45,50-61]. Regardless of injury location, FPI leads to cognitive dysfunction [23,51,55,61,62] and thus it can be a useful model for posttraumatic dementia. Furthermore, FPI delivered laterally is an appropriate model for posttraumatic epilepsy [63].

The FPI model in the rat has been the most widely used model for TBI. Nevertheless, for both CFP and LFP variability's in injury parameters between laboratories are evident. For instance, initial studies using LFP detected an ipsilateral brain injury [64] whereas some later studies detected a widespread, bilateral brain injury [65-67]. One crucial factor determining the outcome severity in this model seems to be the positioning of the craniotomy as already a small shift in the craniotomy site is associated with marked differences in neurological outcome, lesion location and size [68,69]. Thus, establishing a FPI model necessitates extensive methodological fine-tuning to obtain a standardized outcome in respect to its severity and pathophysiology. Once the FPI model is established, the induced brain trauma seems to be highly reproducible.

To enable the use of transgenic mice, Carbonell et al. [49] adapted the FPI model to the mouse. Similar to the rat, the inflicted injury in mice leads to cognitive dysfunction, microglial activation and neuronal and axonal damage [23,49,51,63,70,71].

## Controlled cortical impact injury model

Controlled cortical impact (CCI) models utilize a pneumatic pistol to deform laterally the exposed dura and provide controlled impact and quantifiable biomechanical parameters. This model was adapted to rat in 1991 [72] and to mouse in 1995 [73] and produces graded, reproducible brain injury.

Dependent on the severity of injury, CCI results in an ipsilateral injury with cortical contusion, hemorrhage and blood-brain barrier disruption [74]. Neuronal cell death and degeneration, astrogliosis, microglial activation, inflammatory events, axonal damage, cognitive deficits, excitotoxicity and cortical spreading depressions are reported to ensue [23,26,30,73,75-82]. Particularly with regard to brain edema, CCI is an important model as it presumably causes a cytotoxic and a vasogenic brain edema [23,26,83-89] and thus it reflects the clinical situation of posttraumatic brain edema formation. The predominantly focal brain injury caused by CCI makes this model to a useful tool for studying the pathophysiology of the secondary processes induced by focal brain injury. Interestingly, CCI in rodents is associated with posttraumatic seizure activity similar to the injury-induced epilepsy in humans [90,91]. Thus this model is particularly suitable to study pathomechanisms of posttraumatic epilepsy.

## Cryogenic injury model

The method of cryogenic injury in rodents [92,93] leads to a focal brain lesion. The brain injury in this model is generally produced by applying a cold rod to the exposed dura in rats (e.g. on the parietal cortex using a copper cylinder filled with an mixture of acetone and dry ice (-78°C) [94]) or skull in mice (e.g. on the parietal cortex using a copper cylinder filled with liquid nitrogen (-183°C) [95]). In some studies, a dry ice pellet was directly applied to the skull of the rat or mouse [96,97]. Different injury severities can be achieved by varying the contact time to the exposed cortex [98].

In rodents, cortical cryogenic injury results in a focal brain lesion and breakdown of the blood-brain barrier [94,95]. The primary lesion is surrounded by a penumbral zone where secondary processes lead to an extension of lesion size accompanied by neuronal cell death and cytotoxic and vasogenic edema [98,99]. These secondary processes also include activation of astrocytes and inflammation [95,96,100-103]. Moreover, it was reported recently that a discrete cryogenic lesion to the parietal cortex of juvenile mice causes delayed global neurodegeneration [104]. Due to epileptic activities surrounding the focal lesion, this method is also used for mimicking certain aspects of epilepsy [105-107].

The cryogenic brain lesion model is particularly suited for investigating TBI-associated blood-brain barrier leakage and vasogenic brain edema. However, this focal trauma model lacks the countracoup and diffuse axonal injuries that typically complicate human head injuries [1]. Thus the cryogenic brain lesion model only conditionally mimics the clinical situation. Although various other models reflect more realistic the pathophysiological characteristic of TBI, the cryogenic brain lesion model has one major advantage: The lesions caused by the cryogenic injury model are clearly circumscribed and highly reproducible in size, location and pathophysiological processes of the secondary lesion expansion at the cortical impact site. The high reproducibility of the cortical lesion is particularly useful to screen the impact of pharmacological treatments or gene knockout on secondary lesion development after focal brain injury.

## Other models

### Models to induce diffuse brain injury

In addition to the original Marmarou's weight-drop model, various other impact acceleration models that induce diffuse brain injuries have been described in the literature. As an example, in one model the rat is placed on its back while the head is accelerated upward by a piston [108]. The severity of injury depends on the impact velocity of the piston. In another study, rats were subjected to impact acceleration head injury, using a pneumatic impact targeted to a steel disc centered onto their skull. The animal's head was supported by a soft pad to decelerate the head after the impact [109]. To induce moderate head concussion without focal injury, a pendulum can be used that stroke on the skull midline of rats [110].

### Models to induce focal brain injury

In an attempt to create a model of focal cerebral contusion without diffuse brain injury, Shreiber et al. (1999) generated cerebral contusions and associated evolving damage by a transient non-ablative vacuum pulse applied to the exposed cerebral cortex [111]. Other models designed to generate focal cortical injury inject fluids leading to an inflammatory response and a progressive cavitation [112], apply a mechanical suction force through the intact dura [113] or apply a stab wound [114]. Each of these models result in clearly circumcised focal lesions and thus, similar to the cryogenic injury model, they might be helpful in studies evaluating putative treatments by monitoring the focal lesion size.

### Models to mimic blast-induced neurotrauma

In recent years, exposure to blast is becoming more frequent foremost in military populations. Brain injuries due to blast are caused by particles propelled by blast-force, acceleration and declaration forces and/or the blast wave itself [115]. The non-impact blast injury exhibits an interesting pathophysiology characterized by diffuse cerebral brain edema, extreme hyperemia and a delayed vasospasm [115]. To investigate blast-induced neurotrauma different models have been established. As an example, to mimic a non-impact blast-induced neurotrauma, rodents were fixed and exposed to blast waves caused by detonation of explosive [116] or compressed air [117]. Recently the pathobiology of TBI caused by blast and the animal models for non-impact blast injury have been recently reviewed by Cernak and Noble-Haeusslein [115].

### Combined and modified injury models

If no convenient model is available to address specific research topics, the modification of already existing animal models might be useful. As an example human TBI is often induced by angular (a combination of linear and rotational) accelerations, e.g. TBI caused by car accidents. This clinical scenario was mimicked in rats by instantly rotating the animal to reproduce rotational acceleration after it had sustained the impact that produced linear acceleration using the Marmarou's weight drop model [118]. Another example is the Maryland model, in which Kilbourne et al. mimicked a frontal impact by modifying the impact-acceleration model of Marmarou [119]. To simulate concussions in National Football League players, a rat model was developed in which a pneumatic pressure in the style of CCI models is used to impact laterally the helmet-protected head [120]. Clinical TBI is frequently accompanied by complications such as hypoxic episodes and sepsis. In order to mimic those clinical situations, they can be integrated in the study design (hypoxia [121,122] and sepsis [123]).

## Cell culture models

Cell culture is currently the most promising alternative to animal research. The use of cell culture models simulating TBI might be useful for certain research goals, such as high throughput drug screenings or the assessment of the effect of trauma on individual cell types. The current available cell culture models include models using disruption of various cell cultures by laceration, compression, acceleration or stretch injury (reviewed by [124]).

## Outlook

Initially, the rodent models for TBI were designed to mimic closely the clinical sequelae of human TBI. In this respect, the most straightforward rodent models are the weight-drop models by Marmarou and Shohami as they closely mimic the real life TBI. The inflicted injuries are predominantly diffuse or focal in nature, respectively. Similarly the FPI model and CCI model mimic various injury processes associated with human TBI. Probably due to the excellent reproducibility of induced brain trauma, FPI and CCI are the most widely used rodent models for TBI. However, even small modifications in the experimental design often lead to differences in primary injury and hence to differences in pathobiological processes leading to secondary injury. Considering the heterogeneity of human TBI, scientific hypothesis should be tested in multiple rodent models resulting in distinct types of injury. Thus, models solely mimicking focal or diffuse injury are needed.

In conclusion, there are numerous rodent models of TBI available, widely varying in their ability to model pathomechanisms associated with human TBI. They provide the experimental backbone for investigating TBI pathomechanisms and for the initial testing of neuroprotective compounds.

## Competing interests

The authors declare that they have no competing interests.

## Authors' contributions

CAW drafted the manuscript. ALS corrected and wrote the final manuscript. Both authors read and approved the final manuscript.
